# Multidisciplinary surgery for intravenous leiomyomatosis with inferior vena cava and/or intracardiac extension: a case series

**DOI:** 10.3389/fcvm.2026.1815852

**Published:** 2026-07-15

**Authors:** Feifeng Lin, Xia Liu, Bingqing Huang, Yunhong Lei, Jianjie Huang, Shuo Chen, Qiuling Fan, Zheng Chen, Minghong Shen

**Affiliations:** 1 Department of Gynecology, Shengli Clinical Medical College of Fujian Medical University, Fuzhou University Affiliated Provincial Hospital, Fujian Provincial Hospital, Fuzhou, Fujian Province, China; 2 Department of Cardiovascular Surgery, Shengli Clinical Medical College of Fujian Medical University, Fuzhou University Affiliated Provincial Hospital, Fujian Provincial Hospital, Fuzhou, Fujian Province, China; 3 Department of Obstetrics and Gynecology, Xianyou County General Hospital, Putian, Fujian Province, China; 4 Department of Radiology, Shengli Clinical Medical College of Fujian Medical University, Fuzhou University Affiliated Provincial Hospital, Fujian Provincial Hospital, Fuzhou, Fujian Province, China

**Keywords:** inferior vena cava leiomyomatosis, intracardiac leiomyomatosis, intravenous leiomyomatosis, multidisciplinary, surgery

## Abstract

**Objective:**

The aim of this study was to explore the clinical characteristics, multidisciplinary treatment experience of patients with intravenous leiomyomatosis with inferior vena cava and/or intracardiac extension and propose several surgical tips.

**Methods:**

A retrospective analysis was conducted on the clinical data of 5 patients who were diagnosed with intravenous leiomyomatosis accompanied by inferior vena cava or intracardiac extension between January 2016 and December 2024. The data included demographics, imaging findings, multidisciplinary collaboration, surgical details and outcomes.

**Results:**

The median patient age was 46.0 (41.0–51.5) years. All patients had a history of uterine fibroids or hysterectomy related to uterine fibroids. The most common symptoms included pelvic and/or intracardiac masses (80%), and abdominal distension (20%). All patients had inferior vena cava and iliac vein tumors, 3 (60%) patients had concomitant intracardiac tumors, and 1 (20%) patients had ovarian vein masses. All patients underwent a one-stage surgery, during which the pelvic and vascular/right atrial tumors were completely removed. The volume of blood loss during surgery was 800 (450–4,600) mL. Postoperative complications included pulmonary/pelvic infections (60%), incomplete intestinal obstruction (20%), and lower extremity and iliac vein thromboses (*n* = 1, 20%). The median follow-up time after surgery was 30 (14–74) months, and no recurrence was observed.

**Conclusions:**

Intravenous leiomyomatosis involving the inferior vena cava or heart is relatively rare. Surgery for this type of patient is difficult and may result in significant bleeding. Accurate preoperative evaluation, multidisciplinary treatment, and relevant surgical techniques are crucial for complete tumor resection.

## Introduction

Intravenous leiomyomatosis is a rare benign leiomyoma that grows along veins and is often accompanied by uterine leiomyomas. It usually originates from the pelvic veins and spreads upwards along the venous system, thereby affecting the iliac vein, inferior vena cava, and heart (1, 2). This condition can cause serious clinical consequences and even endanger life in severe cases. The biological behaviour of intravenous leiomyomatosis is unique, as it progresses and grows within the venous channel without invading the venous vessel wall, and some intravenous leiomyomatosis types exhibit jumping growth in the venous system and cardiac cavity, thus representing a special type of uterine leiomyoma (3, 4). Intravenous leiomyomatosis is clinically rare, with diverse clinical manifestations observed depending on the location of tumor involvement. For example, if it involves the inferior vena cava and/or heart, lower limb oedema, chest tightness, shortness of breath and difficulty with breathing may occur, and the clinical consequences can be severe (5).

For intravenous leiomyomatosis with large blood vessels and/or intracardiac extension, complete surgical resection of the tumor is the best treatment choice, as it is crucial for preventing recurrence (6). However, this type of surgical procedure is relatively complex, with a high risk of major bleeding being reported during the surgery. Therefore, preoperative assessment is necessary, and multidisciplinary collaboration (including gynaecology, cardiology, and vascular surgery) is needed during the surgery. Moreover, assisted extracorporeal circulation may be necessary.

Although studies have reported that tumors can be removed through several methods, the optimal surgical method and technique remain unclear because of limited data. Therefore, in this study, we aimed to document the clinical features and multidisciplinary treatment experience of intravenous leiomyomatosis with an inferior vena cava and/or intracardiac extension, along with the associated surgical methods and tips.

## Methods

A total of 15 patients who complained of increased menstrual flow, lower abdominal pain, abdominal distension, and pelvic masses were diagnosed with intravenous leiomyomatosis by gynecological ultrasound and/or enhanced computed tomography (CT) or confirmed by postoperative pathology and were treated at Fuzhou University Affiliated Provincial Hospital between January 2016 and December 2024. Among the 15 patients, 5 (33.3%) were diagnosed with intravenous leiomyomatosis extending to the inferior vena cava. Among the patients, 3 had concomitant right atrial tumors. We retrospectively analysed the information of these 5 patients with intravenous leiomyomatosis extending to the inferior vena cava and/or heart from the electronic medical records database, including patient age, symptoms, clinical features, imaging findings, tumor markers, surgical methods, and treatment outcomes. All patients provided written informed consent, and the study was approved by the Ethics Review Committee of Fuzhou University Affiliated Provincial Hospital (No. K2025-07-008).

### Preoperative multidisciplinary evaluation and discussion

All of the patients underwent detailed preoperative imaging evaluations, including pelvic ultrasound, CT venography or pelvic magnetic resonance imaging (MRI) and echocardiography. Multidisciplinary teams, including gynecological oncologists, cardiovascular surgeons, anesthesiologists and intensive care unit doctors, conducted multidisciplinary assessments and discussions to determine the surgical strategy and perioperative management. The surgical strategy was determined mainly on the basis of the preoperative imaging results. After multidisciplinary evaluation, patients who were deemed capable of complete tumor resection underwent one-stage surgery, otherwise, they underwent two-stage surgery.

### Surgical procedure

All of the patients subsequently underwent one-stage surgery, which was performed via collaboration between the gynecological oncologists and cardiovascular surgical teams. Gynecological surgery combined with inferior vena cava/iliac vessel/cardiac surgery was performed for all of the patients, during which time the pelvic and vascular/right atrial tumors were completely removed, and the bilateral ovaries were removed. The specific surgical details are as follows. After general anaesthesia was successfully achieved, the surgery was performed by utilising total midline laparotomy. The retroperitoneum was opened for identification and ligation of the ovarian vessels. The uterus or residual cervix and pelvic tumors, as well as the bilateral allopian tubes and ovaries, were removed. The inferior vena cava, common iliac vein, and internal and external iliac veins were dissected and exposed. The ureters were freed, and hysterectomy/pelvic mass resection and bilateral adnexectomy were performed. During tumor resection, a longitudinal incision of approximately 2 cm was made on the surface of the left common iliac vein near the inferior vena cava. Using a vascular clamp, the tumor was gradually clamped, and the tumor was removed from the inferior vena cava and left common iliac vein through this incision. The volume of the tumor in the right common iliac vein was too large to be completely extracted through the incision on the surface of the left common iliac vein. Therefore, the extracted tumor was removed first, after which the tumor residue was returned to the right common iliac vein (which was later extracted through an incision in the right common iliac vein). Afterwards, a 4-0 prolene suture was used to continuously suture the vascular incision. The same technique was applied for tumor resection of the right common iliac vein and the variant iliac vein. These vascular incisions were sutured using 5–0 Prolene sutures. Patients with concomitant intracardiac tumors underwent removal of these tumors via an incision from the inferior vena cava, or underwent sternotomy and intracardiac tumor resection with extracorporeal circulation support.

### Postoperative evaluation

All of the surgical specimens underwent histopathological examination, and the diagnosis of intravenous leiomyomatosis extending to the inferior vena cava and/or heart was confirmed by at least two pathologists, combined with imaging and surgical findings.

All of the patients underwent follow-up CT venography 4 weeks after the surgery to evaluate the thoroughness of the surgery. Patients with combined right atrial tumors underwent follow-up cardiac ultrasound after the surgery to determine whether any residual tumor was present in the heart.

### Follow-up

Regular follow-up was conducted every 3–6 months after the surgery was performed; moreover, pelvic examination and pelvic ultrasound were conducted during follow-up. Vascular ultrasound was performed every 6–12 months.

### Statistical analysis

All of the variables are presented as numbers (%) or medians (interquartile ranges). All of the statistical analyses were performed using R, version 4.3.0.

## Results

**Figure 1:**
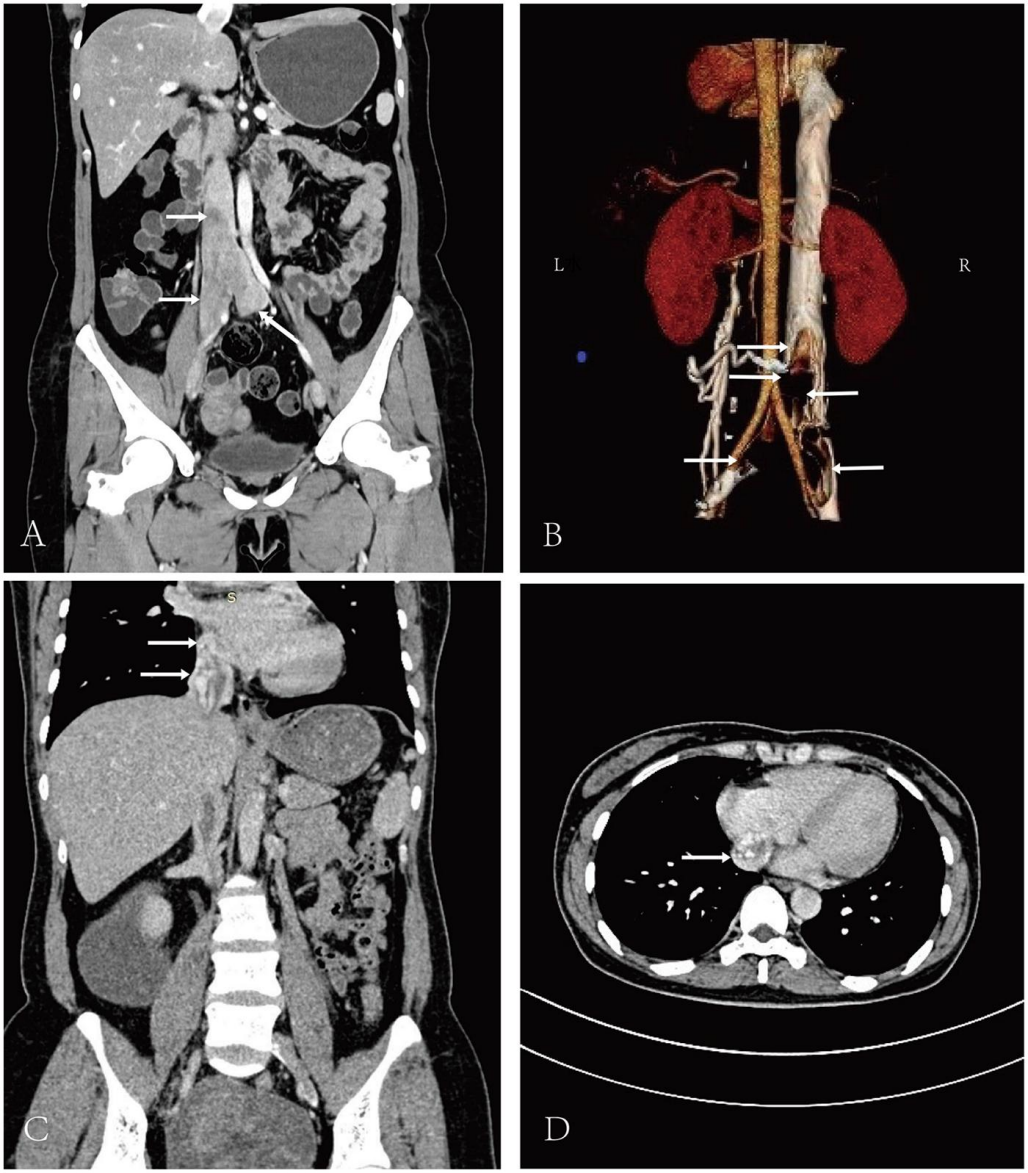
Computed tomography venography image of intravenous leiomyomatosis with inferior vena cava, iliac veins, or intracardiac extension. **(A)** Tumors in the inferior vena cava and bilateral common iliac veins (arrows) in Patient 1. **(B)** Tumors in the inferior vena cava and bilateral common iliac veins presenting filling defects in the corresponding vascular sites (arrows) in Patient 1. **(C)** The tumor in the inferior vena cava extends to the right atrium (arrows) in Patient 5. **(D)** Tumor in the right atrium (arrows) in Patient 5.

**Figure 2:**
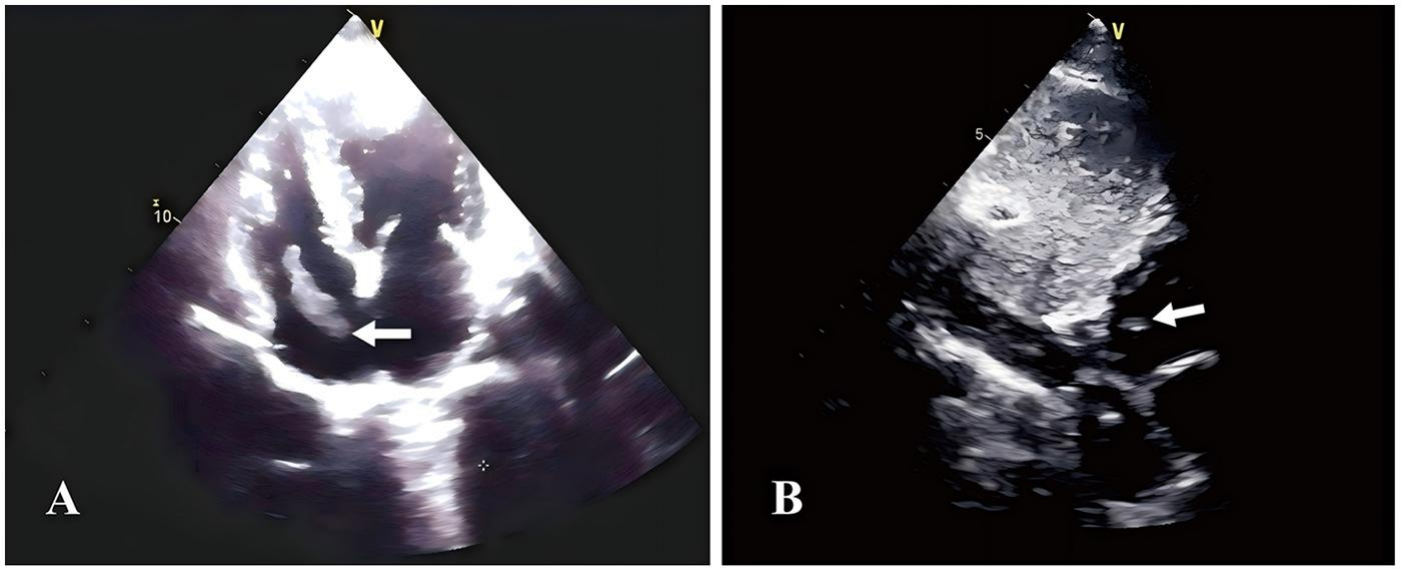
Transthoracic echocardiographic images of intravenous leiomyomatosis with intracardiac extension. **(A)** Apical four-chamber view shows a hypoechoic tumor mass (arrows) in the right atrium of Patient 3. **(B)** Subcostal view demonstrates tumors (arrows) in the right atrium of Patient 5.

Intravenous leiomyomatosis with an inferior vena cava and/or intracardiac extension was present in 33.3% (5/15) of all intravenous leiomyomatosis patients. The demographic and clinical features of the 5 patients are shown in Table 1. The median patient age was 46.0 (41.0–51.5) years. Among the 5 patients, 4 (80%) had a history of uterine fibroids. Among the 4 patients with a history of uterine fibroids, 2 (40%) had previously undergone total hysterectomy or subtotal hysterectomy related to uterine fibroids. The most common symptoms and signs included pelvic and/or intracardiac masses (80%) and abdominal distension (20%). All of the patients had inferior vena cava tumors confirmed by CT venography (Figure 1). Among them, 5 (100%) patients had iliac vein tumors, 3 (60%) patients had concomitant intracardiac tumors confirmed by echocardiography (Figure 2), and 1 (20%) patient had an ovarian vein mass.

**Table 1:** Demographic and clinical features.

Case	Age (years)	Menopause	History of uterine fibroids	History of surgery related to uterine fibroids	Clinical symptoms and signs	Preoperative imaging examination
1	37	Yes	Yes	Subtotal hysterectomy	Asymptomatic pelvic mass	Abdominal ultrasonography Computed tomography venography Pelvic Magnetic resonance imaging Echocardiography
2	45	No	No	None	Asymptomatic pelvic mass	Abdominal ultrasonography Computed tomography venography Echocardiography
3	47	No	Yes	None	Asymptomatic pelvic and intracardiac mass	Abdominal ultrasonography Computed tomography venography Echocardiography
4	56	Yes	Yes	Total hysterectomy	Asymptomatic intracardiac mass	Abdominal ultrasonography Computed tomography venography Echocardiography
5	46	No	Yes	None	Abdominal distension	Abdominal ultrasonography Computed tomography venography Echocardiography

**Figure 3:**
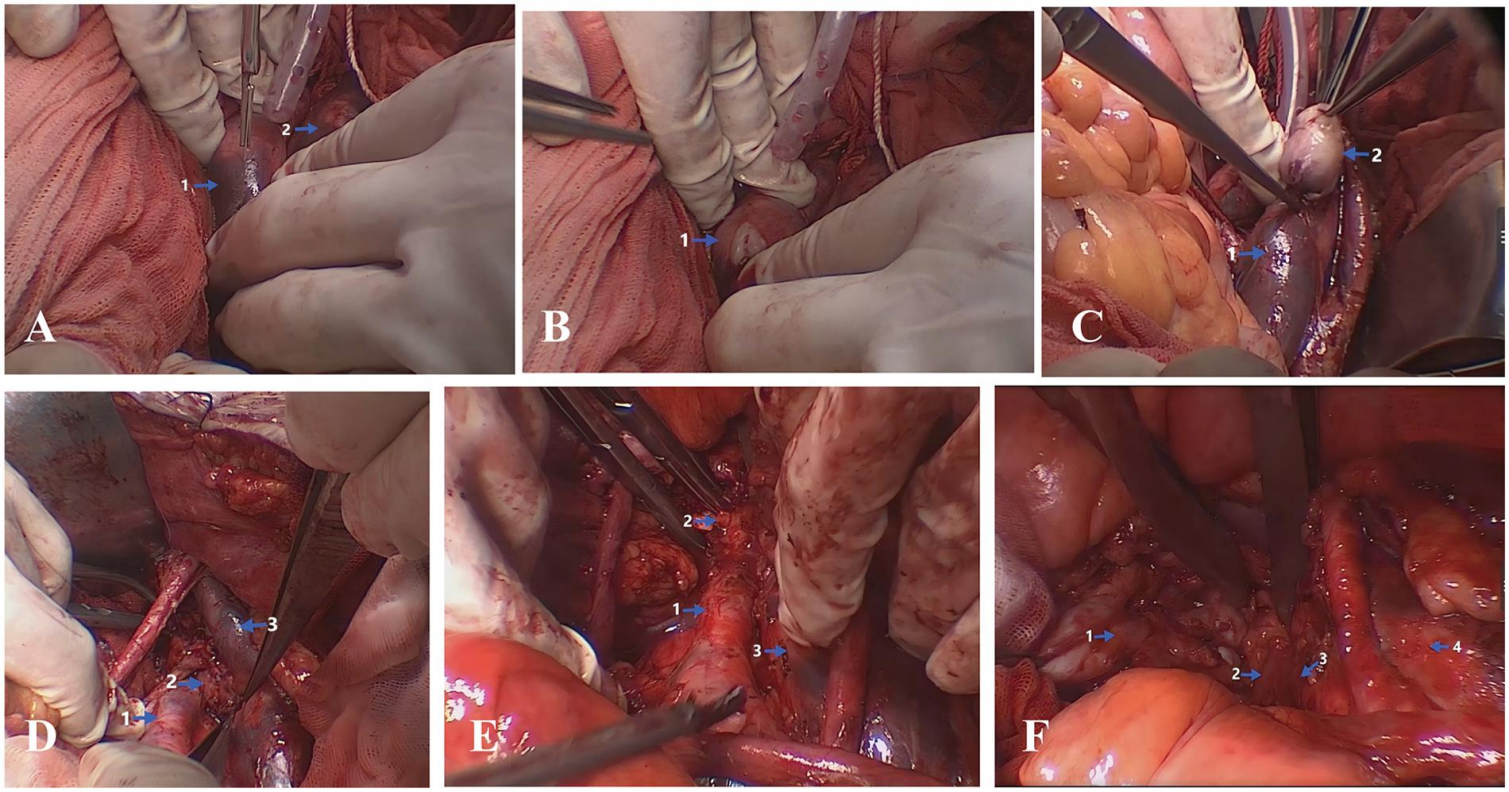
Intraoperative images of patient 1. **(A)** Exposure of the bilateral common iliac veins (1, left common iliac vein; 2, right common iliac vein). **(B)** The left common iliac vein was incised to expose the tumor (1, left common iliac vein). **(C)** Removal of the tumor from the right external iliac vein (1, right external iliac vein; 2, tumor thrombus). **(D)** Variant iliac vein (1, variant iliac vein branching out from the left common iliac vein; 2, right internal iliac vein; 3, right external iliac vein). **(E)** Peripheral vessels of pelvic tumors with tumor thrombi extending to the variant iliac vein (1, variant iliac vein; 2, neovascularization of pelvic tumors; 3, right internal iliac vein). **(F)** Association between pelvic tumors and right iliac vessels (1, pelvic tumors; 2, variant iliac vein; 3, right internal iliac vein; 4, right external iliac vein).

None of the patients received antioestrogen therapy before surgery, and all patients underwent one-stage surgery including inferior vena cava incision tumor resection, pelvic or abdominal mass resection, total hysterectomy or residual cervical resection, and bilateral salpingo-oophorectomy. Among the 3 patients with intracardiac tumors, the intracardiac tumors were removed via an incision of the inferior vena cava (*n* = 2) or sternotomy and intracardiac tumor resection (*n* = 1). For patients with iliac vein or ovarian vein tumors, the tumor in the corresponding vein was removed during the surgical procedure. During the surgery, all of the patients' tumors were completely removed. In all the patients, both the ovaries and the fallopian tubes were removed. Although Patient 1 was only 37 years old, considering her previous history of subtotal hysterectomy related to uterine fibroids, bilateral oophorectomy was performed at the patient's request to reduce the risk of postoperative recurrence. The surgical details and outcomes of the 5 patients are presented in Table 2. The operative time was 280 (235–570) min, and the volume of blood loss was 800 (450–4,600) mL. In Patient 1, who had a history of subtotal hysterectomy, cord-like tumors could be observed in the inferior vena cava, bilateral common iliac veins, right variant iliac vein, and right internal and external iliac veins, with the tumors extending to the proliferating veins of the cervical and pelvic tumors. Five main veins were incised during the surgery, and each incision was accompanied by significant bleeding. Moreover, a significant increase in blood vessels surrounding the pelvic tumors with intravascular tumor thrombus was observed, thus leading to a very difficult tumor removal process. As a result, the volume of intraoperative bleeding reached 8,000 mL. After a considerable amount of blood transfusion, the tumors were ultimately completely removed. The intraoperative image of Patient 1 is shown in Figure 3. For the remaining 4 patients, no more than 3 incisions were made in the major vein area (including the cardiac incision) during surgery, with intraoperative bleeding of 300–1,200 mL being reported.

**Table 2:** Surgical details and outcomes.

Case	Locations of the tumor	Intraoperative Blood Loss (mL)	Operative duration (min)	Intraoperative blood transfusion	Admission to the intensive care unit postoperatively	Postoperative complications	Postoperative length of stay (days)
1	Inferior vena cava Iliac vein Pelvis	8,000	649	Yes	Yes	Pulmonary infection Incomplete intestinal obstruction	21
2	Inferior vena cava Iliac vein Ovarian vein Uterus	800	220	Yes	Yes	Pelvic infection	14
3	Inferior vena cava Right atrium Iliac vein Pelvis	1,200	250	Yes	Yes	Pelvic infection	23
4	Inferior vena cava Right atrium Iliac vein Pelvis	300	280	No	Yes	None	15
5	Inferior vena cava Right atrium Iliac vein Uterus	600	490	Yes	Yes	Lower extremity and iliac vein thromboses	26

Postoperative complications included pulmonary/pelvic infection (*n* = 3, 60%), incomplete intestinal obstruction (*n* = 1, 20%), and lower extremity and iliac vein thromboses (*n* = 1, 20%). Among them, one patient developed multiple thromboses in the right iliac vein and right lower limb after surgery, after which this patient underwent inferior vena cava filter insertion and right lower limb venous catheter thrombolysis.

All of the patients received regular follow-up after the surgery. The median follow-up time was 30 (14–74) months. Because the ovaries of all of the patients were removed during surgery, they did not receive any antioestrogen hormone therapy after the surgery. No recurrence was observed during follow-up.

## Discussion

Intravenous leiomyomatosis is a rare uterine smooth muscle tumor observed in clinical practice and is characterised by the growth of intravenous smooth muscle tumors. Notably, intravenous leiomyomatosis exhibits poor biological behaviour, whereby it grows along vessel walls and extends to the inferior vena cava, or even the right atrium and pulmonary artery. Intravenous leiomyomatosis originating from the uterus often enters the inferior vena cava through the iliac vein or occasionally, it can also enter through the ovarian vein (7, 8). As reported, 56.0–66.7% of intravenous leiomyomatosis cases are accompanied by inferior vena cava and/or intracardiac extension (5, 7). In this study, the incidence of intravenous leiomyomatosis accompanied by inferior vena cava and/or intracardiac extension was 33.3%. The clinical manifestations of intravenous leiomyomatosis combined with inferior vena cava and/or intracardiac extension are diverse and depend on the size and location of the tumor. If mechanical heart obstruction occurs, it can lead to respiratory distress, congestive heart failure, chest pain, and sudden death (in severe cases) (9–12).

Intravenous leiomyomatosis is prone to misdiagnosis or missed diagnosis before surgery (13, 14). Intravenous leiomyomatosis combined with inferior vena cava or intracardiac extension usually occurs in premenopausal women, with an average age of occurrence of 44 years; moreover, it is recognised as being associated with a history of hysterectomy or myomectomy related to uterine leiomyomas (8). A previous study revealed that 38 of 68 intravenous leiomyomatosis patients with intracardiac extension (55.9%) had a history of hysterectomy. It is speculated that intravenous leiomyomatosis of the pelvic vein during hysterectomy was not diagnosed in these patients (2). Similarly, 40% of the patients in this study had a history of surgery related to fibroids. Therefore, for patients with uterine fibroids or a history of uterine fibroid surgery, if pelvic masses, iliac vascular masses, inferior vena cava masses or intracardiac masses are observed, the possibility of this disease should be considered before the surgery. Imaging examinations are helpful for determining intravenous leiomyomatosis and evaluating the size, extent, and severity of the tumors. CT examination (especially CT venography) can help clarify the origin, extension range, and relationship between the venous wall and the tumor. When intravenous leiomyomatosis tumors involve the inferior vena cava, the lumen of the inferior vena cava thickens and filling defects are visible, which present as soft tissue shadows; when intravenous leiomyomatosis tumors involve the heart, a mass can be observed inside of the right atrium or right ventricle, which extends from the mass in the inferior vena cava (15–17). Furthermore, echocardiography may assist in determining the relationship between tumors and venous or intracardiac walls, as well as evaluating the impact of the tumors on intracardiac or intravascular blood flow (18).

Surgery is the most effective treatment for intravenous leiomyomatosis with inferior vena cava or intracardiac extension. The goal of surgery is to achieve complete removal of the tumors. However, because the fact that intravenous leiomyomatosis may involve the bladder, ureter, and lateral pelvic wall in the pelvic cavity, as well as large blood vessels and the heart outside, to provide appropriate treatment decisions and achieve better treatment outcomes, the evaluation and treatment of these patients should be conducted via multidisciplinary collaboration among gynaecologists, vascular surgeons, cardiac surgeons, radiologists, anaesthesiologists, and intensive care physicians. Surgery can be completed in one or two stages. One-stage surgery has several advantages, including the avoidance of haemodynamic complications between the two surgeries, the avoidance of the risk of second general anaesthesia, and the avoidance of the risk of tumor embolism caused by incomplete tumor resection during the one-stage surgery (6, 19). When a preoperative evaluation reveals that inferior vena cava and intracardiac tumors are easy to remove, the patient's physical condition is considered to be good, and the hospital conditions are appropriate, we recommend choosing one-stage surgery as much as possible. Two-stage surgery requires the removal of all of the tumors in two stages. The first stage involves the removal of tumors in the heart and inferior vena cava, whereas the second stage involves the removal of tumors in the pelvic cavity and large vessels. Two-stage surgery may be considered for patients who are in poor condition, who have large tumors, or who cannot simultaneously tolerate multidisciplinary surgery (5). In this study, all of the patients successfully underwent one-stage surgery and had their tumors were completely removed. Pulmonary/pelvic infections (3/5, 60%) and venous thromboses (2/5, 40%) were the most commonly reported postoperative complications. Pulmonary/pelvic infections were considered associated with long-term and large wounds during surgery. Deep vein thrombosis is a possible complication after intravenous leiomyomatosis surgery. A case series reported that the incidence of deep vein thrombosis after intravenous leiomyomatosis surgery was 35.7% (5/14) (20). We speculated that deep vein thrombosis may be associated with a long surgical time and intraoperative blood transfusion.

Several tips are recommended for performing surgery for intravenous leiomyomatosis with inferior vena cava and/or intracardiac extension. For example, multidisciplinary evaluations, including gynecological oncology, cardiovascular surgery, imaging, and the intensive care unit assessments, are crucial before surgery (21). By examining the location of the maximum transverse diameter of the tumor via CT venography, the order of tumor resection during surgery can be guided, and efforts should be made to incise and remove the tumor from this area during surgery, which is helpful for tumor extraction after venous incision. During surgery, the inferior vena cava and iliac vessels should be fully freed and exposed for easy observation, thus resulting in space for vascular occlusion during tumor removal and facilitating potential vascular repair in the future. Typically, tumors can be easily removed through open heart surgery. However, in some patients, open heart surgery may not be necessary. We initially performed open heart surgery on a patient with an intracardiac tumor, but later reported that no adhesion was observed between the intracardiac tumor and the heart wall, thus leading to an easy removal. Therefore, in subsequent cases of intracardiac tumors, the intracardiac tumor was directly removed from an incision in the inferior vena cava. Similar situations have been reported in previous studies (5, 6, 8, 22). Therefore, when the lesion involves the heart (especially when the tumor has a relatively large diameter in the inferior vena cava), an incision can be made through the inferior vena cava, and the tumor can be slowly extracted from the heart through the incision. Furthermore, gentle movements should be emphasised during surgery to prevent unnecessary venous rupture, increased bleeding, and local tissue damage. Because large blood vessels or cardiac tumors usually originate from uterine/pelvic lesions, which are located at the root of the tumor, it is difficult to extract tumors within large blood vessels without first removing the uterine/pelvic tumors. Therefore, we recommend initially cutting the uterine/pelvic tumor before removing the large vessel tumor thrombus, which can reduce the surgical difficulty of large vessel tumors. In patients who have undergone previous hysterectomy, the tumor is prone to adhere to the blood vessel wall, which may make it difficult to remove the tumor (Patient 1). Furthermore, significant intraoperative bleeding may be observed; if necessary, preparations should be made to remove the iliac vessels that are affected by pelvic tumors.

Patients with intravenous leiomyomatosis combined with inferior vena cava and/or intracardiac extension have an increased risk of tumor recurrence after surgery. Studies have reported a recurrence rate of 50% (15/30) in patients with intravenous leiomyomatosis combined with inferior vena cava and/or intracardiac extension during a median follow-up period of 12 months (8). Wang et al. reported a recurrence rate of 27.8% (5/18) over a median follow-up period of 20.5 months (5). Moreover, Lian et al. reported on 10 patients with intravenous leiomyomatosis combined with inferior vena cava and/or intracardiac extension who were followed up for 27–120 months. Except for one case of pelvic leiomyoma recurrence one year after surgery, no recurrence was observed in the iliac vein or inferior vena cava (23). In this study, no recurrence was observed in any of the patients, with a median follow-up of 30 months being implemented.

Owing to the rarity of intravenous leiomyomatosis, current studies are mostly in the form of case series or case reports; thus, the risk factors associated with recurrence are still unclear. However, one study revealed that compared with patients who underwent complete resection, those who underwent incomplete tumor resection demonstrated a higher postoperative recurrence rate (50% vs. 21.4%, *P* = 0.016), indicating a close relationship between incomplete tumor resection and tumor recurrence (5). Another study revealed that complete tumor resection, age, cardiac involvement, choice of one-stage or two-stage surgery, choice of either bilateral oophorectomy or postoperative hormone therapy, and the duration of hormone therapy (> 6 months or ≤ 6 months) were not significantly correlated with postoperative recurrence (7). The risk factors associated with tumor recurrence require further clarification.

The advantage of this study is that it not only elucidated the clinical and imaging characteristics of intravenous leiomyomatosis combined with inferior vena cava and/or intracardiac extension but also presented videos, surgical techniques, and precautions for such complex patients. However, this study has several limitations. Owing to the rarity of intravenous leiomyomatosis combined with inferior vena cava and/or intracardiac extension, the number of patients involved in this study was small, and there were no related cases of recurrence, making it impossible to conduct further analysis on tumor recurrence.

Owing to the complexity of the surgery and the high risk of massive bleeding during the surgery, surgical treatment of intravenous leiomyomatosis with inferior vena cava and/or intracardiac extension remains challenging. Multidisciplinary cooperation and surgical skills are very important. In this study, we presented and analysed the clinical characteristics, multidisciplinary treatment, and surgical techniques for intravenous leiomyomatosis combined with inferior vena cava and/or intracardiac dilation, which may provide reference data for the clinical diagnosis and treatment of such complex cases.

## Conclusions

Intravenous leiomyomatosis involving the inferior vena cava or heart is relatively rare. Surgery for this type of patient is difficult and may result in significant bleeding. Accurate preoperative evaluation, multidisciplinary surgery, and relevant surgical techniques are crucial for complete tumor resection.

## Data Availability

The original contributions presented in the study are included in the article/Supplementary Material, further inquiries can be directed to the corresponding authors.
